# μCT of *ex-vivo* stained mouse hearts and embryos enables a precise match between 3D virtual histology, classical histology and immunochemistry

**DOI:** 10.1371/journal.pone.0170597

**Published:** 2017-02-08

**Authors:** Christian Dullin, Roser Ufartes, Emanuel Larsson, Sabine Martin, Marcio Lazzarini, Giuliana Tromba, Jeannine Missbach-Guentner, Diana Pinkert-Leetsch, Dörthe M. Katschinski, Frauke Alves

**Affiliations:** 1 Institute for Diagnostic and Interventional Radiology, University Medical Center, Goettingen, Germany; 2 Synchrotron Light Source ‘Elettra,’ Trieste, Italy; 3 Department of Molecular Biology of Neuronal Signals, Max Planck Institute of Experimental Medicine, Goettingen, Germany; 4 Center Nanoscale Microscopy and Molecular Physiology of the Brain (CNMPB), Goettingen, Germany; 5 Clinic for Haematology and Medical Oncology, University Medical Center, Goettingen, Germany; 6 Institute of Cardiovascular Physiology, University Medical Center, Goettingen, Germany; University of Notre Dame, UNITED STATES

## Abstract

The small size of the adult and developing mouse heart poses a great challenge for imaging in preclinical research. The aim of the study was to establish a phosphotungstic acid (PTA) ex-vivo staining approach that efficiently enhances the x-ray attenuation of soft-tissue to allow high resolution 3D visualization of mouse hearts by synchrotron radiation based μCT (SRμCT) and classical μCT. We demonstrate that SRμCT of PTA stained mouse hearts ex-vivo allows imaging of the cardiac atrium, ventricles, myocardium especially its fibre structure and vessel walls in great detail and furthermore enables the depiction of growth and anatomical changes during distinct developmental stages of hearts in mouse embryos. Our x-ray based virtual histology approach is not limited to SRμCT as it does not require monochromatic and/or coherent x-ray sources and even more importantly can be combined with conventional histological procedures. Furthermore, it permits volumetric measurements as we show for the assessment of the plaque volumes in the aortic valve region of mice from an ApoE-/- mouse model. Subsequent, Masson-Goldner trichrome staining of paraffin sections of PTA stained samples revealed intact collagen and muscle fibres and positive staining of CD31 on endothelial cells by immunohistochemistry illustrates that our approach does not prevent immunochemistry analysis. The feasibility to scan hearts already embedded in paraffin ensured a 100% correlation between virtual cut sections of the CT data sets and histological heart sections of the same sample and may allow in future guiding the cutting process to specific regions of interest. In summary, since our CT based virtual histology approach is a powerful tool for the 3D depiction of morphological alterations in hearts and embryos in high resolution and can be combined with classical histological analysis it may be used in preclinical research to unravel structural alterations of various heart diseases.

## Introduction

The development of advanced imaging techniques for phenotyping the heart and circulatory system in small animal models has provided novel insights into cardiovascular pathophysiology [1]. To pursue this research avenue new developments are necessary to combine high resolution organ imaging with the characterization of the analysed tissue. Despite intensive advancements in the field of imaging, traditional histology remains the gold standard for morphological tissue assessment. Histological analysis is well established, and allows the depiction and discrimination of various tissue and cell types as well as extracellular matrix at high spatial resolution. Furthermore, immunohistochemistry (IHC), by utilizing labelled protein specific antibodies, enables the visualization of protein expression such as disease related biomarkers within the tissue slide. However, analysing the heart by histology and IHC provides only planar information about the sample and the loss of tissue as well as implementation of artefacts due to the cutting procedure leads to a fragmentary depiction [2]. Most importantly, once embedded in paraffin, the orientation of the cutting planes is fixed and cannot be easily modified. By contrast, X-ray based CT is a true 3D technique, which can be applied in a large spatial range down to submicron resolution [3] and therefore appears particularly interesting for a virtual histology approach of the mouse heart. Data derived from an overlay of CT imaging and histology would therefore benefit novel information from combined high resolution imaging and morphological analysis. CT imaging of soft-tissue like the heart muscle or vessels produces poor contrast, but which can be enhanced by applying staining solutions or contrast agents containing heavy ions such as iodine, barium or tungsten [4]. We used phosphotungstic acid (PTA), a solution containing tungsten ions [5], which are known to bind to fibrin and collagen [6]. PTA, among other contrast agents such as Lugol, has recently been used for high-contrast imaging of insects and embryonic tissues of mouse and chicken at histological resolutions using commercial μCT systems [7–9].

Here, we report the feasibility of synchrotron radiation based μCT (SRμCT) and classical μCT followed by histological staining and immunohistochemical procedures to assess the morphology of PTA stained embryonic and adult mouse hearts in 3D and in great detail. We show that volume measurement of plaque formation in the aortic valves of ApoE^-/-^ mice and the 2D depiction of virtual cut sections of regions of interests can be performed.

## Materials and methods

### Animal models

ApoE^-/-^ mice fed a high fat diet are a common mouse model of atherosclerosis [10]. The inactivation of the ApoE gen results in an increased total cholesterol level which leads to the development of atherosclerotic plaques, an effect that can accelerated by using a high fat diet. Hearts were excised from healthy adult (22 weeks old) female C57BL6/N mice as well as from ApoE^-/-^ mice and their control littermates (obtained from Harlan Laboratories), both fed for 70 days with a high fat diet (Kliba Nafag, 2126, 45% kcal % fat) ad libitum. In addition to the atherosclerosis model, embryos and postnatal C57BL6/J mice were sacrificed at the ages E12, E15, E18, P0, P2, P5 and analysed in toto. All animal in vivo procedures were performed in compliance with the guidelines of the European (Directive 2010/63/EU) and the German ethical laws and were approved by the administration of Lower Saxony (Nds. Landesamt für Verbraucherschutz und Lebensmittelsicherheit (LAVES), Dezernat 33 –Tierschutzdienst), Germany {33.9-42502-04-12/0705}. The ex-vivo analysis of the mouse embryos was as well approved by the administration of Lower Saxony, Germany {33.9-42502-04-10/0314}.

### Sample preparation and PTA staining

Excised hearts of adult mice were briefly rinsed five times in water and then transferred to 35% ethanol and 70% ethanol for 1 hour each. For staining and fixation, hearts were placed for 6 days at room temperature (RT) under slow rotation in a 4% paraformaldehyde solution (PFA, Serva Electrophoresis) in phosphate-buffered saline, pH 7.4 (PBS, Invitrogen), containing 0.7% phosphotungstic acid solution (PTA, Sigma-Aldrich Corp.) diluted in 70% ethanol. For storage, samples were briefly rinsed in water and transferred to fresh 70% ethanol. For further μCT analysis, the PTA stained hearts were removed from 70% ethanol and embedded in either 1% agarose (Carl Roth GmbH + Co. KG) in 1.8 ml vials (Nunc CryoTube Vials, Sigma-Aldrich Corp.) or in paraffin blocks (Suesse Labortechnik) after an ascending ethanol series as described in Martin et al. [11]. An unstained heart of an adult female C57BL6/J mouse fixed in 4% PFA only was used for comparative analysis.

Whole mouse embryos (E12, E15, E18) and whole bodies of mice at the postnatal stages P0, P2 and P5 were PTA stained using a modified protocol. Embryos were briefly rinsed five times in water and then stained in a solution containing 0.7% PTA diluted in 70% ethanol for 6 hours, each at 4°C under slow rotation. Then, embryos were fixed and stained for 4 to 11 days (see Table 1, list of incubation times for the different developmental stages) at RT under slow rotation in a 4% PFA solution in PBS, pH 7.4, containing 0.7% PTA diluted in 70% ethanol. All juvenile mice from E18 onwards were skinned to facilitate PTA penetration. All samples were embedded in 1% agarose gel in plastic tubes that were appropriate for the samples size to ensure sample stability during the CT acquisition. In order to assess the shrinkage of the mouse hearts induced by the PTA staining approach, 10 adult C57BL6/J mice were sacrificed and the hearts explanted. Subsequently, they were scanned with the in-vivo μCT (QuantumFX, Perkin Elmer), followed by PTA staining and paraffin embedding as described above and scanned again after each procedure with the same in-vivo μCT.

**Table 1:** Samples analysed.

	N	system used	duration of PTA staining to achieve full penetration [d]
excised hearts–wild type	6	SRμCT	6
excised hearts–ApoE -/-	5	eXplore Locus SP	6
embryo—E12	14	Nanotom	4
embryo–E15	20	SRμCT	5
embryo–E18	8	SRμCT	6
postnatal–P0	8/5	Nanotom/SRμCT	7
postnatal–P2	16	SRμCT	9
postnatal–P5	16	Nanotom	12
excised hearts—C57BL6/J	4	QuantumFX	6

The postnatal and adult mice were sacrificed using carbon dioxide (CO_2_). The mouse embryos were euthanized by immersion in ice water, carefully keeping their head above the water surface.

### CT scanning and reconstruction

For high resolution SRμCT, the phase contrast CT beamline SYRMEP at the Synchrotron Light Source “Elettra” (Trieste, Italy) was applied [12]. Images of all samples were reconstructed using the standard filtered backprojection (FBP) algorithm implemented in the SYRMEP tomo project (STP) developed by the SYRMEP group [13]. Hearts from the arteriosclerosis mouse model were analysed using the eXplore Locus SP μCT (developed by GE Healthcare and serviced by TriFoil). Some of the PTA stained embryos were scanned with the Nanotom specimen μCT (Phoenix, GE Measurement & Control). The QuantumFX in-vivo μCT (Perkin Elmer) was used to assess the shrinkage of the excised hearts after the PTA staining and paraffin embedding process. Sample data sets can be found at (osf.io/8nmm2).

### Histological procedures

Following the CT scans, the excised, PTA stained hearts were further processed for histology and IHC. Heart samples were manually removed from the agarose gel, chemically dried using an ascending ethanol series and embedded in paraffin (Suesse Labortechnik). No pre-processing steps (such as chemical drying and embedding) were needed for the PTA stained heart that was already embedded in paraffin. All samples were then cut in 2 μm thick sections and stained with haematoxylin and eosin (HE) [14] or with Masson-Goldner-Trichrome (MGT) as described before [15]. For IHC, sections were deparaffinised, hydrated, and boiled for 10 min in 10 mM citrate buffer (pH 6.0), followed by cooling for 20 min at RT. Sequential blocking (Avidin/Biotin/Fish block) for 10 min each was used to prevent unspecific antibody binding. Staining was performed with a rat monoclonal antibody directed against the endothelial cell marker CD31 (clone SZ31, diluted 1:10) for 30 min at RT. Subsequently, sections were incubated with an anti-rat-biotinylated secondary antibody (BioLegend, diluted 1:200) for 1 h at RT, followed by detection with avidin-horseradish-peroxidase (eBioscience, diluted 1:400), for 1 h at RT. The sections were then counterstained with haematoxylin. Images were obtained with an Axioskop microscope (Zeiss) equipped with a digital camera (Micropublisher 5.0, QImaging Surrey).

### Data analysis

The software Scry (v5, Kuchel & Sautter GbR) was used for 3D visualization and quantification of the data sets. The Wilcoxon signed-rank test was performed in PAST (folk.uio.no/ohammer/past).

## Results

### μCT imaging allows precise 3D imaging of ex-vivo PTA stained mouse hearts

**Fig 1:**
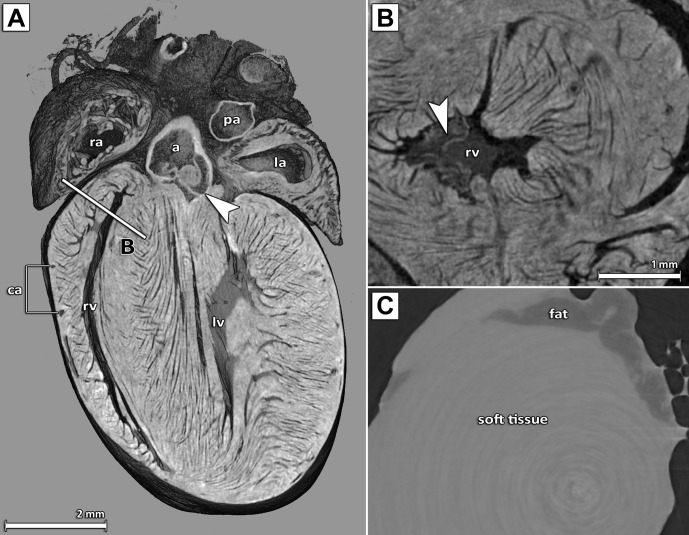
SRμCT results of excised PTA stained and agarose gel embedded mouse hearts. (A) Virtual cut through a volume rendering representation of a PTA stained and agarose gel embedded excised heart of an adult mouse scanned by SRμCT. The coronal cut displays details of the anatomical structures: the right atrium (ra), the left atrium (la), the right and left ventricle (rv, lv), some coronary arteries (ca) as well as the aorta (a) and the aortic valve (white arrow head) and the pulmonary artery (pa). In addition, the PTA staining allows for identification and representation of the orientation of the muscle fibre bundles. (B) Detailed view of the PTA stained right ventricle shown in (A). The position and orientation of the virtual cut section shown here is indicated by the line labelled B in panel (A) demonstrating that virtual sections are feasible at any position and in any orientation. (C) A representative image of the right ventricle area of an unstained heart, which shows no contrast with the exception of a visible difference between fatty and soft-tissue. Note, due to the absence of contrast, (C) may represent a slightly different region than (B).

In order to assess the feasibility of μCT imaging to visualize hearts ex-vivo in great detail, hearts were excised from healthy female adult mice (Table 1) and stained with PTA for 6 days before embedding in agarose. SRμCT imaging was performed with parameters outlined in Table 2. A virtual cut through a volume rendering representation of a PTA stained and agarose gel embedded excised heart is shown in Fig 1A and 1B and demonstrates that a homogenous staining of the soft-tissue within the heart was achieved. Soft-tissue such as myocardium, epicardium and endocardium can effectively be discriminated by SRμCT due to their differences in PTA uptake. Anatomical structures of the heart such as aorta, left and right atrium, left and right ventricle as well as fibres of the heart muscle and the valves are clearly delineated. Due to the fact that cavities and blood vessels are not stained by PTA they appear as dark areas. By comparison, SRμCT of the unstained heart does not allow delineation of the cavities, muscle fibres and/or vessels, showing only a faint difference between fatty and soft-tissue (Fig 1C). In order to demonstrate that the approach is not limited to SRμCT, explanted mouse hearts stained in the same way were also scanned with a classical benchtop μCT (eXplore Locus SP) (S1 Fig). The obtained images differ in image quality, but comparable structures can be seen in both samples, scanned either with SRμCT or with classical μCT. In order to quantify the diffusion speed, one heart of an adult mouse was scanned daily during the staining process using the low dose in-vivo μCT QuantumFX (Perkin Elmer) with the parameters summarized in Table 2. We found an average PTA diffusion speed of about 20 μm/h in the first 24 hrs, which then reduced to a constant diffusion speed of about 10 μm/h in the following days (data not shown).

**Table 2:** Acquisition parameters.

	stained sample PTA_aga_	stained sample PTA_par_	unstained sample UST	arteriosclerosis mouse model	mouse embryos	excised hearts for shrinkage measurement
embedded in	agarose gel	paraffin	-	agarose gel	agarose gel	-/-/paraffin
system / technique	SRμCT	SRμCT	SRμCT	eXplore Locus SP	Nanotom	QuantumFX
ring energy [GeV]	2.4	2.0	2.0	-	-	-
x-ray photon energy [keV]	28	24	17	-	-	-
tube parameter	-	-	-	60kVp/100μA	100kVp/100μA	90kVp
rotation angle [°]	180	180	180	360	360	360°
projections	1800	1200	900	1800	1800	3600
detector exposure time [s]	1.2	1.2	0.75	7	0.75	0.03
detector binning	2x2	4x4	4x4	1x1	2x2	2x2
voxel size [μm^3^]	9x9x9	18x18x18	18x18x18	8x8x8; 16x16x16‡	5x5x5; 10x10x10†	40x40x40

‡ different voxel sizes were used either for the entire heart (16μm) or to measure the plaque volume (8μm).

† different voxel sizes were used depending on the size of the embryo.

### Virtual histology of whole PTA stained mouse embryos and postnatal mice allows 3D analysis of heart development

**Fig 2:**
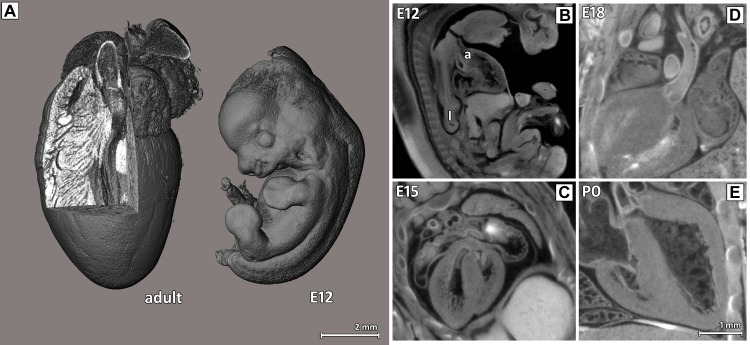
Virtual cut sections through the heart region of PTA stained mouse embryos and postnatal mice. (A) The volume rendering of adult mouse heart and entire mouse embryo at E12 demonstrate that the adult mouse heart has approximately the same size as the entire mouse embryo at E12. Virtual transvers sections are shown for E12 (B), E15 (C) and E18 (D) and P0 (E) displaying details of the anatomical structures: the aorta (a) and lungs (l). As early as E12, the heart shows nearly the final shape and structure, as do other organs like the lung, and later only increase in size. Two different CT systems were used: classical μCT for E12 and SRμCT for E15, E18 and P0, demonstrating that with both CT techniques good and comparable results were obtained.

In order to investigate mouse heart development, mouse embryos at different ages E12, E15, E18 and postnatal mice at P0 and P5 were PTA stained, embedded in agarose and imaged ex vivo using either SRμCT or classical μCT as outlined in Table 1. In comparison, a PTA stained adult mouse heart embedded in agarose which had the same size as the whole embryo at E12, is shown in Fig 2A. However, in the absence of any soft-tissue staining no sufficient contrast in CT imaging can be achieved as shown in S2 Fig.

Although the cardiovascular system is the earliest functioning organ system of the mammalian embryo, growth and differentiation of the heart can be depicted at different developmental stages by imaging of PTA stained mouse hearts with μCT (Fig 2B–2E). The aorta of the heart at E12 with a heart size of 2 mm can be clearly discriminated (Fig 2B). The septum is completely closed and loose myocardium does not show muscle bundles yet. Ventrally the heart is confined by a strongly accentuated diaphragm. The small dense lung between columna and heart is well visible. Fig 2C shows the image of a E15 heart in which the atria are notably well depicted. Section of the aorta and the closed septum are delineated, the myocardium is dense and thick, although muscle bundles are still not identifiable. The small dense lung is noticeable. A transverse digital virtual section of the E18 heart in Fig 2D demonstrates that density and thickness of the myocardium increases at this time point. As PTA stains especially the septum wall and the wall of the aorta, both structures are discernible. Fig 2E shows a transversal virtual section of the P0 heart. Epicardia (Pericardia) are clearly depicted and the aortic valve and pulmonary valve are defined. Single tendinous chords connecting the papillary muscles with the tricuspid valve are clearly visible due to their high content of collagen (80%) which is stained by PTA. Note, that the air filled alveoli of the lung are also evident around the heart. Movies of virtual cuts through virtual histology data sets of exemplary mouse embryos (E12, E15, E18) and postnatal mice (P0 and P5) are presented in S1–S5 Movies.

### CT based virtual histology of PTA stained samples still allows subsequent IHC and enables the definition of virtual cutting planes that precisely match classical histology

**Fig 3:**
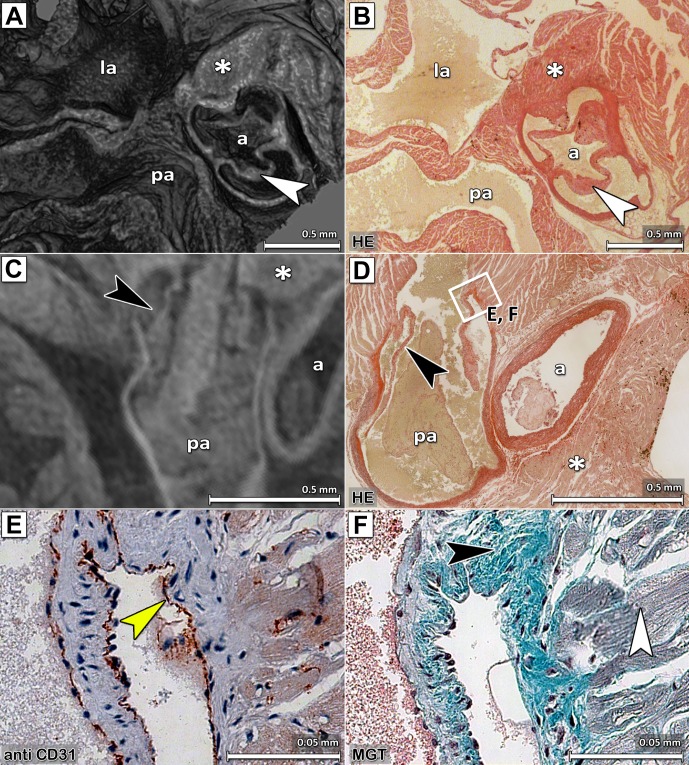
Comparison of CT data sets with histological procedures. Virtual cross sections through the SRμCT data sets of a PTA stained and (A) agarose gel embedded or (C) paraffin embedded excised mouse heart and their corresponding histological sections (B) and (D) are shown. Notably, even very small structures like the flaps of the aortic valves (indicated by white arrow heads) can be seen by SRμCT. The following structures are clearly depicted: a, aorta; pa, pulmonary artery; la, left atrium and star, heart muscle. HE staining of (B) a section of a heart scanned in agarose gel and later embedded in paraffin and (D) a section from a heart embedded in paraffin before CT acquisition, demonstrates that heart morphology is preserved for classical histology after PTA staining and SRμCT scanning. The pulmonary artery of the heart in the SRμCT image (C) shows a faint PTA contrast, in correlation with the finding of blood residues in the histological section (D). Beside the fact that the CT data of the paraffin embedded heart (C) shows less contrast and more noise, the aortic valves are still clearly depicted (black arrow head). (E) and (F) present results of histological sections adjacent to the one shown in (D) at the position indicated by the white rectangle. (E) Immunostaining with an anti-CD31 antibody of the heart that was CT scanned in paraffin demonstrates the feasibility of IHC on PTA stained heart sections and shows the endothelium indicated by a yellow arrow. (F) MGT staining of a heart embedded in paraffin that was CT scanned visualized collagen in green (black arrow) and smooth muscle cells in violet (white arrow).

As current μCT devices cannot provide the same specific contrast as IHC and classical histology, the combination of virtual histology with IHC or conventional histology would provide invaluable new information. Therefore, we determined whether conventional histological methods can still be performed on samples that were PTA stained and imaged with μCT. To this end, tissue sections of scanned hearts were further processed for HE, MGT staining and IHC. Fig 3 shows representative virtual images obtained by SRμCT of an agarose embedded (Fig 3A) compared with a paraffin embedded heart (Fig 3C) and their corresponding histological heart sections (Fig 3B and 3D–3F). These results demonstrate that PTA stained samples already embedded in paraffin can be scanned by μCT with comparable image quality. As in this approach no re-positioning or re-embedding of the sample prior histology is needed a precise correlation between CT and histology can be ensured.

Both techniques (μCT and histology) deliver anatomical structures such as pulmonary artery, left atrium and aorta of the heart in great detail (Fig 3A and 3C). MGT staining of the sample embedded in paraffin after CT acquisition revealed intact collagen (green) and muscle (red) fibres (Fig 3F) demonstrating that MGT can be used in combination with the PTA staining. Positive staining of CD31 on endothelial cells by IHC, indicated by yellow arrows on Fig 3E, illustrates that the CD31 epitope of the sample was not influenced by the PTA staining and SRμCT.

2D conventional histology is irreversibly defined by the cutting plane. Due to the 3D nature of the SRμCT imaging approach, virtual sections through the heart can be calculated at any position and in any orientation. In order to demonstrate that SRμCT and histological data can be overlaid, a histological HE stained section of the aortic valve was chosen and the 3D model of the CT data was virtually cut and rotated to display the corresponding CT section through the heart (Fig 3A and 3C). Main anatomical features of the heart such as vessel walls and valves are clearly visible in both, the SRμCT and the histological data sets (Fig 3B and 3D). Moreover, even small structures like the flaps of the valves of both, the aortic or pulmonary arteries, can be illustrated by SRμCT (Fig 3A and 3C, white and black arrowheads, respectively) and were confirmed by classical histology (Fig 3B and 3D, white and black arrowheads, respectively). The potential of such an approach is demonstrated in S3 Fig showing that a virtual slice through a CT data set of the paraffin embedded heart (S3A Fig) can be overlaid with the image of a H&E stained slice of the same sample at the same location (S3B Fig) with only minor deviations (S3C Fig).

In order to validate that our PTA staining protocol does not introduce additional shrinkage of the tissue, hearts of 10 wildtype C57BL6/J were scanned with an in-vivo μCT i) directly after excision, ii) after PTA staining, 6 days post explantation and iii) after being embedded in paraffin. Heart volumes were accessed in 3D following threshold based segmentation. PTA staining does slightly but significantly affect the heart volume, since hearts increased in volume of 8.9% ± 2.3% (average ± standard deviation, p < 0.01 Wilcoxon test) after 6 days of PTA staining. The embedding process on the other hand, which includes the same chemical drying step as used for normal histology, significantly reduced the heart volumes up to 7.1% ± 5.0% (average ± standard deviation, p < 0.01 Wilcoxon test). We thus show, that conventional histological procedures as well as IHC can successfully be applied on sections of PTA stained and SRμCT scanned heart samples regardless of whether they were embedded in agarose gel (Fig 3B) or in paraffin (Fig 3D).

### Virtual histology of PTA stained hearts allows three dimensional localisation and volume measurement of arteriosclerotic plaques in an ApoE^-/-^ arteriosclerosis mouse model

**Fig 4:**
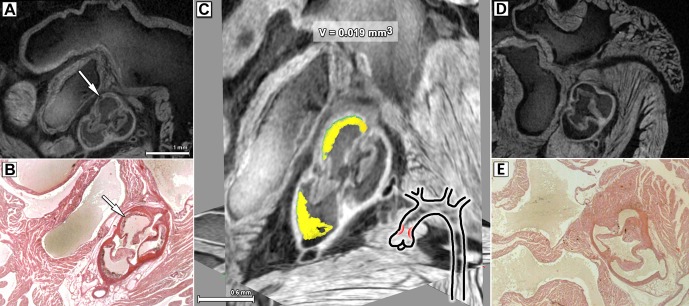
Volume measurement of arteriosclerotic plaques. (A) shows a virtual cross section in the CT data sets of a PTA stained arteriosclerotic heart (ApoE^-/-^ mouse) obtained with classical μCT. A double contour of the vessel wall in the aortic valve (white arrow) can be clearly seen, indicating the fibrotic cap of the plaques as verified by a HE staining of the same heart presented in (B) confirmed the presence of the arteriosclerotic plaque. (C) Three orthogonal slices of the aortic valve region are shown. The arteriosclerotic plaque was segmented in 3D (yellow) and its volume was measured (V = 0.019 mm^3^). In the pictogram the location of the plaque formations is indicated in red. (D) shows the virtual cross section in the CT data sets of a PTA stained heart of a control mouse obtained with classical μCT and (E) shows the corresponding HE staining. No plaque formation can be observed in the areas corresponding to those shown in (A) and (B).

In order to proof the applicability of the μCT-based histological approach in combination with PTA staining to assess aortic plaque formation, we analysed hearts of 12 weeks old female mice fed for 70 day with a high fat diet from the well described ApoE^-/-^ arteriosclerosis mouse model. As shown in Fig 4A, the PTA staining protocol highlights the heart muscle and the fibrotic capsule of the arteriosclerotic plaques in the aortic valve (white arrow), but not the plaques themselves. Therefore, in contrast to the healthy animals which developed no plaques (Fig 4D and 4E), the aortic valves show double contours. The plaque volume was assessed by region growing segmentation in the void space between the double contours (Fig 4A). The virtual histology approach revealed a heterogeneous distribution of plaques in the region of aortic valves of ApoE^-/-^ mice with volumes of 0.02 ±0.02 mm^3^, N = 5. In all wild type controls analysed that have been fed with the same high fat diet for 70 days, no plaques within the aortic valves were identified. HE staining of the paraffin section of the heart (Fig 4B) excised from the ApoE^-/-^ mouse presented in Fig 4A verified the existence of the plaques and the fibrotic cap of the aortic valve of the mouse heart. In contrast, HE staining of the paraffin section of the heart (Fig 4E) of the control mouse, presented in Fig 4D, shows the absence of plaques in the same regions of the heart. Since the explanted heart specimens did not contain the entire aortic arch, the presence or absence of plaques could not be verified.

## Discussion

With this study we demonstrate the feasibility of CT driven virtual histology by SRμCT and classical CT of PTA stained soft-tissue samples for the 3D assessment of mouse hearts and embryos. SRμCT of PTA stained mouse hearts provides detailed images of the cardiac atrium, ventricles, myocardium and dense mechanical heart vessel walls. Furthermore, growth and anatomical changes in structures such as the myocardium, aortic wall, septum and heart valves can be depicted during distinct developmental stages by CT imaging of PTA stained mouse embryos. We show that this approach is not limited to synchrotron radiation by presenting comparable results with classical μCT systems, which are more available to researchers. In addition, we demonstrate that i) our virtual histology approach does not introduce an additional shrinkage of the tissue, ii) can be applied to samples already embedded in paraffin and iii) allows a subsequent analysis by both, classical histology and even IHC.

Compared to other 3D techniques like MRI and medical ultrasound, CT usually offers a higher spatial resolution, which can reach sub-micron values. Alternative CT imaging techniques like phase contrast CT can increase the contrast to noise ratio up to a factor of 200 [16]. While phase contrast CT can thus be employed even for unstained samples, reaching incredible high contrast-to-noise ratios, as demonstrated by others before [17–19], it is practically only feasible at synchrotron light sources and systems using fine focus x-ray tubes. By contrast, our PTA staining approach can be used in combination with any CT system by increasing the absorption based x-ray contrast [7–9]. However, as expected due to the higher brilliance of synchrotron light sources, the image quality of the data sets acquired with SRμCT demonstrates a superior image quality even with voxel sizes larger than the once used for classical μCT.

PTA is known to bind to collagen and fibrin [20] which explains the clear depiction of the heart muscle fibre structures shown here. This technique may thus greatly assist the study of heart fibrosis or modelling the electrophysiological and elastic properties of the heart. This in turn would promote knowledge of structural changes that occur within the organ following a heart attack [21] and would be essential to test novel approaches for replacements [22].

The use of heavy ion based contrast agents including PTA to increase the soft-tissue contrast in CT based virtual histology has already been reported for various applications, ranging from comparative morphology and developmental biology studies in mice, chicken embryos and insects [7–9], to high-throughput phenotyping techniques for mouse embryos [23] and the 3D analysis of post-mortem mouse brains [11]. However, the approach presented here is new, in that it demonstrates that SRμCT and classical μCT in combination with our PTA staining protocol is compatible with subsequent classical histochemical methods like HE and MGT staining as well as IHC, as demonstrated by successful CD31 staining of endothelial cells. In our opinion, this is essential, as it allows to confirm and to extend the 3D virtual histology results by precise identification and correlation of different cell types depicted by histological procedures to anatomical structures visualized by CT, something which is not yet feasible by conventional CT alone. In addition, we demonstrate that by PTA staining of hearts from an ApoE^-/-^ arteriosclerosis mouse model, arteriosclerotic plaques in the aortic valve region can not only be precisely localized in 3D but their volumes can also be quantified. In contrast, precise volume measurements based only on histological analysis is challenging because multiple sections have to be analysed and the cutting process often introduces additional artefacts [2]. The use of ex-vivo μMRI without additional staining for 3D volume measurement of plaque formation in mice as reported by McAteer et al [24] suffers from the limited spatial resolution of 47×47×62.5 μm^3^.

Moreover, we show for the first time that PTA stained samples can be scanned even after hearts were embedded in paraffin, although with a slightly decreased image quality than that achieved for agarose gel embedded samples, due to higher x-ray attenuation and minor scattering introduced by the paraffin. However, this effect is only minor and can be neglected. Paraffin embedding before CT scan has the advantage that no re-embedding for further histological analysis is required, avoiding any additional deformation or shrinkage of the sample and ensuring a 100% correlation of the CT images and histological sections. CT scanning of PTA stained hearts may allow in future to guide the cutting process to obtain histological section of regions of interest within the heart. In all other applications such an overlay image is very difficult as the shrinkage induced by the dehydration process prior to histology does not occur homogenously in a sample containing several tissue types, but rather as a non-ridged deformation as analysed by Schulz et al [25]. Furthermore, we show that the PTA staining protocol itself does not introduce a substantial shrinkage. On the contrary they slightly increased in volume by 8%. Only the chemical drying procedure prior paraffin embedding causes the observed shrinkage of approximately 7%. This however is exactly the same process used for non PTA stained tissue prior histological examination. Therefore, we can ensure that the results obtained in PTA stained hearts are not vary from standard histological examinations.

We demonstrate that even samples of the size of whole mouse embryos and postnatal mice can be PTA stained and analysed by CT, allowing the examination of the anatomy of the hearts in their physiological context and in an intact shape. We found a PTA diffusion speed of about 20 μm/h in the first 24 hrs that reduced to a constant diffusion speed of roughly 10 μm/h. This, together with the fact that we only achieved complete penetration of the postnatal mice by omitting the fixative PFA from the PTA solution in the first 3 hours of staining, suggests that the PFA in the staining solution penetrates the entire sample much faster than PTA, resulting in an increased tissue stiffness that is harder to penetrate by PTA. This is also supported by Helander et al [26] who reported that diffusion speeds for fixatives correlate with the square root of the fixation time. In case of formalin, a depth of about 14 mm would be penetrated within 16 hrs. We thus assume that the total PTA staining time could be reduced if PFA fixation is only applied after full penetration of the tissue sample has been achieved. It still needs to be tested if such an approach provides tissue at a quality that allows for subsequent IHC analysis.

As the diffusion time of the PTA solution depended on the diameter of the sample and had already reached 12 days for the 5 days old mice, the feasibility to stain whole adult mice still has to be explored. Because the PTA staining time is largely affected by the low diffusion speed of heavy ions [7], replacing PTA by a staining solution containing metal ions with a lower atomic number and therefore greater diffusion speed such as iodine may facilitate the staining of larger samples. Such an approach was demonstrated by Degenhardt et al [27], who used Lugol at various concentrations to stain mouse embryos. However, a fast diffusion rate also increases the risk that tissue interfaces appear blurry, due to the only minor differences in the tissue specific diffusion rates and associated small contrast gradient. In addition, PTA has an approx. 2.7 times higher x-ray attenuation value than Lugol at the used x-ray energy of around 28 keV. In this respect, PTA would be preferable because it produces a higher contrast, thus allowing high resolution CT analysis, which is usually hampered by low contrast to noise ratio.

Another approach that may lessen the effect of reduced diffusion rates due to the increased sample stiffness after fixation, could be whole body perfusion with PTA or Lugol as described by Dunmore-Buyze et al.[28]. In their application, the staining time was reduced to 30 min, producing a different staining pattern in dependence of the contrast agent used. However, subsequent IHC was not performed by the authors. Here, whole body perfusion was achieved by opening the chest and injecting contrast agents into the left ventricle of the heart, which is contra-indicated when analysing the heart as in our case.

In summary, we demonstrate that the combination of PTA staining with both, Synchrotron based or classical μCT, allows virtual histology of entire mouse hearts as well as of postnatal mice and embryos in 3D and in great detail and permits histological procedures such as HE and MGT staining as well as IHC. Arteriosclerotic plaques that develop in the ApoE-/- arteriosclerosis mouse model can not only be localised in the aortic valves but also quantified by volume measurements. We believe to have conclusively shown that this virtual histology approach is a powerful tool for the simultaneous high resolution depiction of morphological alterations in 3D and immunohistochemical analysis of the same heart samples. In future, virtual histology data sets will allow for precise localisation and orientation of the tissue or cells in focus in order to guide the cutting process before performing classical histology. Thereby, this method may be used to characterize pathological heart conditions in disease mouse models or address the response to therapy in preclinical research.

## Supporting information

S1 Fig Comparison of SRμCT and classical microCT. Two explanted PTA stained and agarose gel embedded hearts of adult mice are shown. The left heart was scanned by SRμCT with a reconstructed voxel size of 9x9x9 μm^3^ and the right heart was scanned using the specimen microCT eXplore Locus SP with a reconstructed voxel size of 16x16x16 μm^3^. Despite the differences in image quality similar results can be obtained with both imaging techniques.
(TIF)

S2 Fig Comparison of microCT scans of PTA stained and unstained mouse embryos. A) μCT scans of a PTA stained (left) and an unstained (right) mouse embryo (E12) performed with the in-vivo μCT QuantumFX (Perkin Elmer) with a reconstructed voxel size of 40x40x40 μm^3^ are shown with identical visualization settings. In the unstained embryo no details in morphological structures can be discriminated. B) shows cross sections of the same embryos displayed in (A). Also here no details are visible in the unstained embryo.
(TIF)

S3 Fig Overlay of microCT and histology data. A) depicts a virtual cut section through a PTA stained and paraffin embedded heart scanned with SRμCT. B) shows the corresponding H&E stained histological section. C) fusion of the CT and histology is shown illustrating that both data sets can be overlaid with only minor deviations allowing the correlation of histological findings with the localization within the original sample in 3D.
(TIF)

S1 Movie Animated virtual cut sections through a CT data set of a PTA stained mouse embryos (E12). (AVI)

S2 Movie Animated virtual cut sections through a CT data set of a PTA stained mouse embryos (E15). (AVI)

S3 Movie Animated virtual cut sections through a CT data set of a PTA stained mouse embryos (E18). (AVI)

S4 Movie Animated virtual cut sections through a CT data set of a PTA stained postnatal mouse (P2). (AVI)

S5 Movie Animated virtual cut sections through a CT data set of a PTA stained postnatal mouse (P5). (AVI)
