# Supplementary figures and images for: μCT of ex-vivo stained mouse hearts and embryos enables a precise match between 3D virtual histology, classical histology and immunochemistry
Source: PLoS One. 2017 Feb 8;12(2):e0170597. doi: 10.1371/journal.pone.0170597 (PMC5298245; doi:10.1371/journal.pone.0170597)

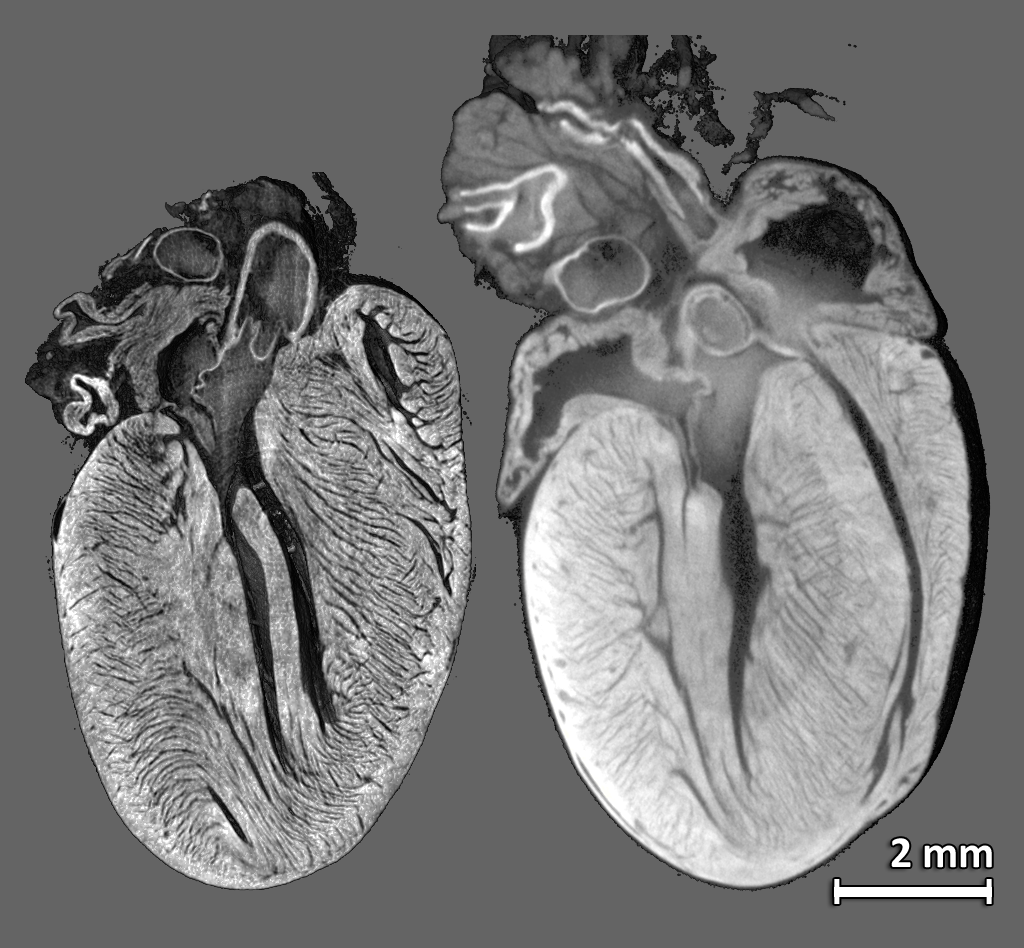

Supplement: S1 Fig — Two explanted PTA stained and agarose gel embedded hearts of adult mice are shown. The left heart was scanned by SRμCT with a reconstructed voxel size of 9x9x9 μm3 and the right heart was scanned using the specimen microCT eXplore Locus SP with a reconstructed voxel size of 16x16x16 μm3. Despite the differences in image quality similar results can be obtained with both imaging techniques. (TIF) [file pone.0170597.s001.tif]

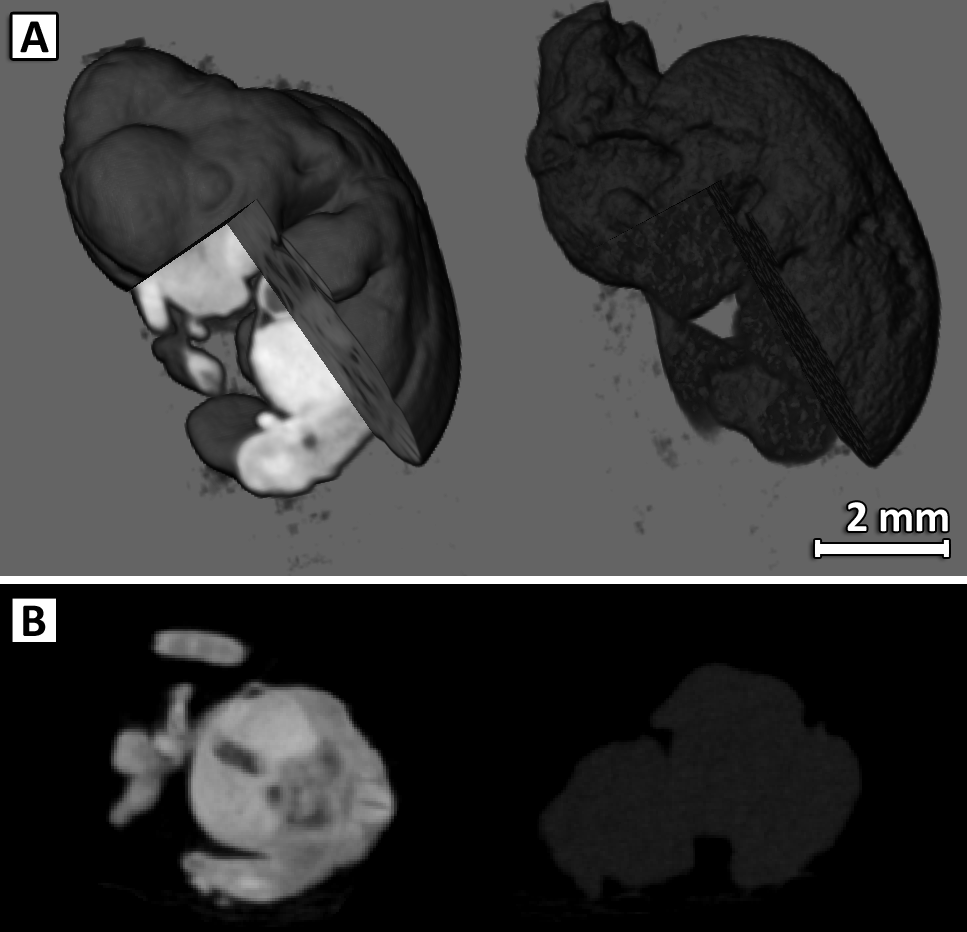

Supplement: S2 Fig — A) μCT scans of a PTA stained (left) and an unstained (right) mouse embryo (E12) performed with the in-vivo μCT QuantumFX (Perkin Elmer) with a reconstructed voxel size of 40x40x40 μm3 are shown with identical visualization settings. In the unstained embryo no details in morphological structures can be discriminated. B) shows cross sections of the same embryos displayed in (A). Also here no details are visible in the unstained embryo. (TIF) [file pone.0170597.s002.tif]

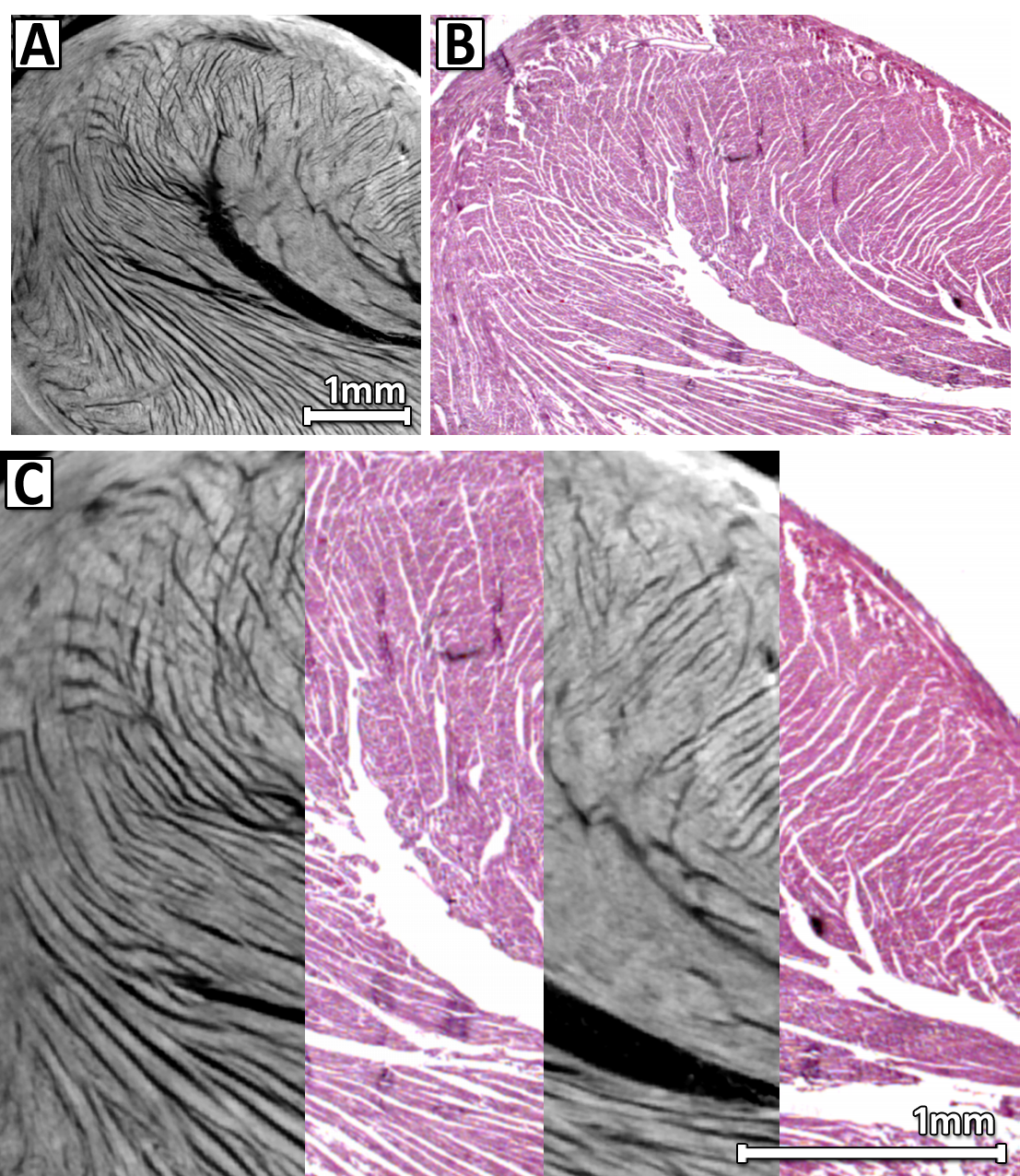

Supplement: S3 Fig — A) depicts a virtual cut section through a PTA stained and paraffin embedded heart scanned with SRμCT. B) shows the corresponding H&E stained histological section. C) fusion of the CT and histology is shown illustrating that both data sets can be overlaid with only minor deviations allowing the correlation of histological findings with the localization within the original sample in 3D. (TIF) [file pone.0170597.s003.tif]
